# Metropolitan Hospital Sunday Fund

**Published:** 1893-08-05

**Authors:** 


### METROPOLITAN HOSPITAL SUNDAY FUND.

A MEETING of the Council was held at the Mansion House on Tuesday last, Sir Sydney Waterlow in the chair. This is regarded as the business meeting of the year, at which the Distribution Committee reports its decisions and members of the Council devote themselves to criticism and inquiry.

Sir SYDNEY WATERLOW had to report his regret at the falling off of upwards of £5,000 in the amount to be distributed as compared with the previous year. He held this falling off to be due to a diminished power on the part of people to give more liberally, and not to any want of interest in the welfare of the hospitals. Trade was not flourishing, people were losing rather than making money, which made all the difference to the charities. Sir Sydney congratulated the Council on the control exercised over the accounts of the hospitals, such control being in the best interests of the institutions themselves, as well as for the protection of the public. There were at present a good many vacant beds in London hospitals, and the public would be well advised to contribute more income to enable them to be fully and continuously occupied, rather than to give large donations to extension funds for the purpose of erecting new buildings. The best managed hospitals had already adopted the uniform system of accounts formulated by the Council after conference with the secretaries. Certain of the institutions published one form of report and balance-sheet and sent another to the Distribution Committee. A letter had in consequence been addressed to each institution which had not yet adopted the uniform system, pointing out the dangers attending their present method and requesting compliance with the Council's requirements. Failing this, it would no doubt be necessary next year for the Distribution Committee to name each institution which has not adopted the system, a step which must lead to the withdrawal of the award. It was satisfactory to know that the uniform system had already been generally adopted.

Mr. THOMAS BRYANT, P.R.C.S., hoped that the fund would refuse assistance to all hospitals which made collections in the streets on their own behalf by means of boxes or processions, as well as to any institution which was declared to be conducted in the interest of any one private person. One man, or private, hospitals were a danger and a nuisance.

Dr. GLOVER, in a brief but forcible address, suggested that a special committee should be appointed to consider how far the Council could tighten its hands upon the management of certain hospitals. He thought that no hospital at which the patients were not treated by a representative medical staff should receive a grant. He also inquired whether the idea of a Central Hospitals Board, suggested by the Lords' Committee, was likely to be established, as he held it would fulfil several useful functions.

Sir SYDNEY WATERLOW, in reply, said the Distribution Committee had done their best to check abuses, and the improper expenditure of the funds of certain hospitals. The reforms must be gradually introduced, and it must be remembered that voluntary institutions were entitled to much consideration, and the right to manage their own affairs. If the affairs of an institution were not properly managed, he would advise the authorities not to come to the fund for a grant. The Distribution Committee discountenanced street collections by certain hospitals, and were determined to give such institutions only one-third of the sum they would otherwise be entitled to. If a Central Hospitals Board was to be effective, it must have behind it the power of the purse; hence the committee referred to by Dr. Glover had recommended the establishment of such a board, with due regard to this important feature. The hospital authorities seemed to object to such a plan, but he thought that they lost sight of the sound reasons upon which it was based.

Mr. BURDETT, in view of his experience of similar action in the United States, deprecated the exclusion of certain hospitals and institutions from grants, providing that they complied with the rules of the fund. He congratulated the Council upon the publication this year of the percentage of the cost of management upon the total expenses, as well as the percentage upon the cost of maintenance only. The proposal to establish a Central Hospitals Board was still under discussion, and he was informed that a meeting of the Special Committee would be called so soon as Parliament broke up, and the chairman, Lord Sandhurst, had time for the work in which he took the deepest interest. Progress had meanwhile been made, as the opponents of the scheme referred to by Sir Sydney Waterlow had drafted one of their own. It was fairly open to debate whether such a board would render material service to the hospitals or the public, but it was satisfactory to know that the questions at issue would shortly be fully debated and settled on their merits.

A list of the grants made to the various hospitals will be found on another page.

Resolutions conveying the thanks of the Council to the Distribution Committee, the Lord Mayor, and the Press, were duly proposed and passed, and the proceedings terminated.

Mr. Howard Potter (Brown, Shipleys, and Co.), an eminent American, who was present, expressed gratified surprise that the Sunday Fund in London not only raised money, but actively interested itself in the well-being, management, and progress of all the hospitals.

